# The Novel Navigation‐Assisted Surgery Increases Financial Burden of Patients Suffering Tile Types B and C Pelvic Fractures in China

**DOI:** 10.1155/bmri/8030943

**Published:** 2025-10-28

**Authors:** Jian Xing, Hao-Ran Li, Wei Wei, Kun Zhang, Ya-Hui Fu, Shuang Han, Peng-Fei Wang, Yan Zhuang

**Affiliations:** ^1^ Department of Orthopedics and Comprehensive Traumatology, Honghui Hospital, Xi′an Jiaotong University, Xi′an, Shaanxi, China, xjtu.edu.cn

**Keywords:** conventional surgery, financial burden, navigation-assisted surgery, pelvic fracture

## Abstract

**Background:**

Studies demonstrating whether patients with pelvic fractures can benefit from robotic technology in terms of financial burden are lacking. This study was aimed at comparing the financial burden of navigation‐assisted and conventional surgeries for Tile Types B and C pelvic fractures in northwest China.

**Methods:**

In total, 188 patients treated at our hospital were included, of whom 140 were treated with navigation‐assisted surgery and 48 with conventional surgery. General, clinical, and economic parameters were compared between the groups. Subgroup analysis stratified by year was performed in the navigation‐assisted surgery group.

**Results:**

Fees for pharmaceutical, nursing, and postoperative hospital stay were significantly higher in the conventional surgery group, whereas fees for anesthesia, surgery, total cost, and surgical duration were higher in the navigation‐assisted surgery group. According to the subgroup analysis, fewer patients were treated in the intensive care unit, and more patients suffered severe injuries over time.

**Conclusion:**

Compared with conventional surgery, navigation‐assisted surgery was associated with lower fees for pharmaceutical, nursing, and postoperative hospital stays and higher fees for anesthesia, surgery, total cost, and surgical duration. The proportion of pharmaceutical costs and nursing fees, as well as the shorter postoperative recovery time associated with navigation‐assisted surgery, may have a positive impact on patients′ overall economic burden.

**Summary**

• Our research compared the financial burden of navigation‐assisted and conventional surgeries for Tile Types B and C pelvic fractures in northwest China.
• In total, 188 patients were included.
• General, clinical, and economic parameters were collected from medical record.
• Navigation‐assisted surgery was associated with lower fees for pharmaceutical, nursing, and postoperative hospital stay and higher fees for anesthesia, surgery, total cost, and surgical duration.
• The proportion of pharmaceutical costs and nursing fees and the shorter postoperative recovery time associated with navigation‐assisted surgery may have a positive impact on patients′ overall economic burden.

## 1. Introduction

Pelvic fractures generally occur in young patients who sustain a high‐energy injury and in the elderly who sustain a trivial injury [1], and they are associated with considerable morbidity and mortality [2]. Surgical treatment is suggested for unilateral or bilateral instability of the posterior or anterior pelvic ring, according to the Tile classification [3–5]. Effective fixation of the pelvic posterior ring determines its integrity and stability and benefits rehabilitation and other treatments of the injured parts [6]. There are many surgical methods for the fixation of different injuries to the posterior pelvic ring. Currently, percutaneous sacroiliac screw fixation appears to be the best solution for restoring the stability of the pelvic posterior ring [7–9]. C. Kim and J. Kim [10] compared plate fixation and sacroiliac screw fixation in the treatment of posterior pelvic ring disruption in a systematic review and meta‐analysis. They reported that the plate group exhibited a longer incision, more blood loss, and a longer hospital stay, whereas the sacroiliac screw group had more x‐ray exposure, more frequent implant loosening, and higher Majeed functional scores.

However, percutaneous sacroiliac screw fixation is the optimal method for the treatment of pelvic posterior ring injury owing to its advantages, including less surgical injury, fewer complications, faster recovery, and better curative effects [11]. The complex anatomy of the posterior pelvic ring and narrow and long sacral bodies result in more x‐ray exposure, a longer operation time, a higher rate of implant position error, and nerve injury, which limit the wide application of percutaneous sacroiliac screw fixation [8]. However, three‐dimensional (3D) navigation system‐assisted percutaneous sacroiliac screw fixation is easier to perform than percutaneous sacroiliac screw fixation. Li et al. reported that the precision and safety of 3D navigation‐assisted surgery were significantly higher than those of conventional surgery [12].

The use of this new technological approach has increased exponentially in orthopedic surgery, as more evidence has presented its utility and the advantages of minimally invasive surgical approaches, such as fast recovery, shorter hospital stays, and less blood loss [13]. Nevertheless, these new and emerging tools have increased concerns about healthcare costs, and cost‐effectiveness considerations are becoming crucial in the debate that supports the use (or not) of robotic technology [14]. Previous studies have reported that robotic methods cost one‐third more than other minimally invasive surgeries for gynecological diseases [13, 14]. Currently, there is no research demonstrating whether patients with pelvic fractures can benefit from robotic technology in terms of financial burden. Therefore, this study was aimed at comparing the difference in costs between navigation‐assisted surgery and conventional surgery for Tile Types B and C pelvic fractures in northwest China.

## 2. Methods

### 2.1. Study Design and Population

Clinical patients diagnosed with pelvic fractures classified as Type B or C according to the Tile classification were collected retrospectively from our hospital from January 2017 to January 2022. Patients diagnosed with acetabular fractures or open fractures and those who received conservative therapy were excluded. Patients treated with navigation‐assisted surgery were grouped into the navigation‐assisted surgery group. Patients treated with conventional surgery, such as the insertion of sacroiliac screws with or without radiographic monitoring, were included in the conventional surgery group.

### 2.2. Data Collection

General data, including age and sex, indicators of personal economic burden, and indicators of clinical data of the included cases, were collected from patients′ medical records. Fracture classification and severity of trauma were assessed by two independent, experienced doctors according to the Tile classification and Injury Severity Score (ISS).

### 2.3. The Definition of Each Cost

Pharmaceutical cost: All pharmaceutical costs incurred by the patient during hospitalization, excluding the costs of anesthetic drugs used during surgery. Bed cost: Bed usage fee during the patient′s hospitalization. Nursing fee: All nursing‐related expenses incurred during the patient′s hospitalization. Inspection fee: All expenses incurred by the patient during hospitalization for laboratory tests, including those for blood tests, respiratory system‐related tests, and other such examinations. Anesthesia fee: Total anesthetic cost during the patient′s surgery, including the cost of anesthetic drugs. Cost of surgery: The surgical fee includes the labor cost for the surgery, general equipment usage fees (including the usage fee for the robot navigation system), one‐time consumable costs (including the one‐time consumable costs for the robot navigation system), such as gauze, iodophor, and instrument sterilization fees, and nursing fees in the operation room and the cost of internal fixation. Blood transfusion expense: Cost of blood products, such as packed red blood cells, fresh frozen plasma, and human serum albumin. Physiotherapy fee: Physical therapy fees including lower limb air pressure pump therapy, postoperative rehabilitation guidance for the affected limb provided by rehabilitation specialists, and auxiliary rehabilitation treatments.

All costs in this study were recorded in the total expense list of patients during their hospitalization.

### 2.4. Statistical Analysis

We only analyzed complete data for the development and verification of the model. Missing data was excluded prior to the statistical analysis. Quantitative data are expressed as median (M, minimum to maximum), whereas age is presented as mean ± standard deviation. Quantitative data were compared using the Student test (*t*‐test) or a nonparametric test, depending on whether the data fit a normal distribution. Qualitative data were compared using the chi‐square test. The multivariate analysis of variance (MANOVA) was used for the correlations between total cost and the included parameters by multiple linear regression. A subgroup analysis stratified by year was performed for the navigation‐assisted surgery group. At *p* < 0.05, differences were considered statistically significant.

### 2.5. Ethical Approval of Studies and Informed Consent

Informed consent was obtained from all participating adult subjects and from parents or legal guardians for minors or incapacitated adults by oral.

## 3. Results

**Figure 1:**
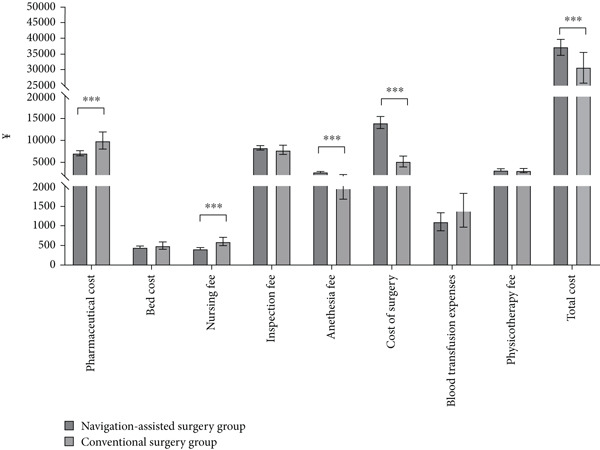
Comparison of economic parameters between the navigation‐assisted surgery and conventional surgery groups.  ^∗∗∗^ *p* < 0.001.

**Figure 2:**
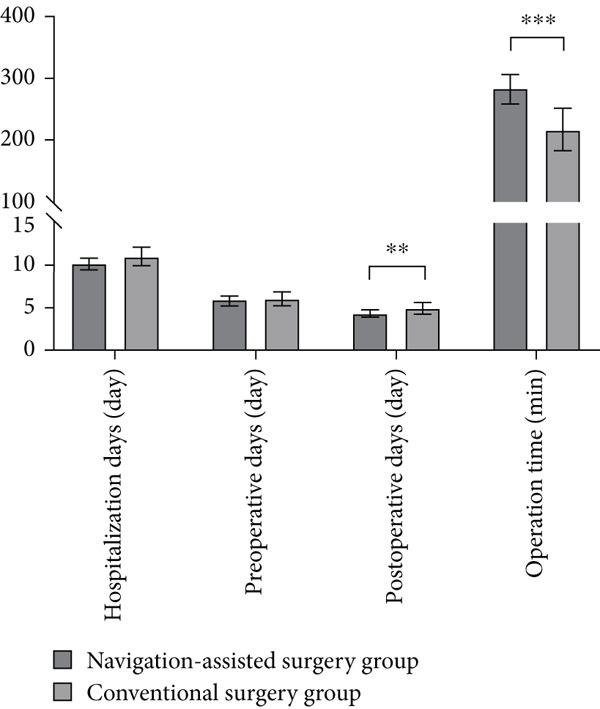
Comparison of the clinical parameters between the navigation‐assisted surgery and conventional surgery groups.  ^∗∗^ *p* < 0.01.  ^∗∗∗^ *p* < 0.001.

In accordance with the inclusion and exclusion criteria, 278 patients were included, 90 of whom were excluded: 40 patients with acetabular fractures, 35 patients with open fractures, and 15 patients treated with conservative therapy. A total of 188 patients were included, of which 140 were assigned to the navigation‐assisted surgery group and 48 to the conventional surgery group. There were no significant differences between the navigation‐assisted surgery and conventional surgery groups in terms of baseline information (Table 1). The navigation‐assisted surgery group had a higher anesthesia fee (M, ¥2375.00 vs. ¥1670.00, *p* < 0.001), cost of surgery (M, ¥10,420.00 vs. ¥4453.00, *p* < 0.001), and total cost (M, ¥35,216.95 vs. ¥25,404.75, *p* ≤ 0.001), whereas the conventional surgery group had a significantly higher pharmaceutical cost (M, ¥6044.49 vs. ¥7081.16, *p* = 0.002) and nursing fee (¥343.00 vs. ¥509.50, *p* < 0.001) (Figure 1 and Table 2). There were no significant differences between the groups in terms of the bed cost (*p* = 0.71), inspection fee (*p* = 0.37), blood transfusion expenses (*p* = 0.11), and physiotherapy fee (*p* = 0.91) (Figure 1 and Table 2). The length of postoperative hospitalization was significantly shorter in the navigation‐assisted surgery group than in the conventional group (M, 4.00 vs. 4.50 days, *p* = 0.02), and the operative time was longer in the navigation‐assisted surgery group than in the conventional group (M, 250 vs. 180 min, *p* < 0.001) (Figure 2 and Table 3). There were no significant differences between the navigation‐assisted surgery group and the conventional surgery group in terms of hospitalization (*p* = 0.06) and preoperative days (*p* = 0.24) (Figure 2 and Table 3).

**Table 1:** Baseline characteristics of the two groups.

	**Navigation-assisted surgery group (** **n** = 140**)**	**Conventional surgery group (** **n** = 48**)**	**p** **value**
Sex, female, *n* (%)	61 (43.57)	23 (47.92)	0.60
Age (years), mean ± SD	47.74 ± 14.23	37.90 ± 14.27	0.97
Surgical method			0.095
Sacroiliac screw fixation+ORIF (Stoppa approach), *n* (%)	89 (63.6)	27 (56.3)	
Sacroiliac screw fixation+ORIF (Kocher–Langenbeck approach), *n* (%)	3 (2.1)	1 (2.1)	
Sacroiliac screw fixation+Infix, *n* (%)	38 (27.1)	10 (20.8)	
Sacroiliac screw fixation+ORIF (ilioinguinal approach), *n* (%)	10 (7.1)	10 (20.8)	
ICU treatment			0.97
Yes, *n* (%)	6 (4.3)	2 (4.2)	
No, *n* (%)	134 (95.7)	46 (95.8)	
Injury severity score			0.05
< 16	71 (50.7%)	34 (70.8%)	
≥ 16	56 (40.0%)	12 (25.0%)	
≥ 25	13 (9.3%)	2 (4.2%)	

Abbreviations: ICU, intensive care unit; ORIF, open reduction and internal fixation.

**Table 2:** Comparison of economic parameters between the navigation‐assisted surgery and conventional surgery groups.

	**Navigation-assisted surgery group (** **n** = 140**)**	**Conventional surgery group (** **n** = 48**)**	**Mann–Whitney** **U** **test result**	**Z**	**p** **value**
Pharmaceutical cost, M (min–max), ¥	6044.49 (1930.63–23,127.08)	7081.16 (3198.96–36,139.31)	2363.00	−3.06	0.002
Bed cost, M (min–max), ¥	400.00 (40.00–1200.00)	400.00 (50.00–1600.00)	3239.50	−0.37	0.71
Nursing fee, M (min–max), ¥	343.00 (38.00–1231.00)	509.50 (66.00–1973.00)	2057.00	−4.01	≤ 0.001
Inspection fee, M (min–max), ¥	7341.00 (1855.50–22,554.00)	7063.35 (1571.00–22,729.40)	3067.00	−0.90	0.37
Anesthesia fee, M (min–max), ¥	2375.00 (1480.00–6550.00)	1670.00 (1125.00–5580.00)	1152.00	−6.79	≤ 0.001
Cost of surgery, M (min–max), ¥	10,420.00 (480.00–33,418.00)	4453.00 (480.00–25,516.00)	1179.00	−6.71	≤ 0.001
Blood transfusion expenses, M (min–max), ¥	620.00 (0.00–6960.00)	1100.00 (0.00–6880.00)	2843.00	−1.61	0.11
Physiotherapy fee, M (min–max), ¥	3075.00 (186.00–8760.00)	2795.75 (373.30–10,299.50)	3321.500	−0.12	0.91
Total cost, M (min–max), ¥	35,216.95 (8825.05–77,941.02)	25,404.75 (7334.25–110,219.21)	2277.00	−3.33	0.001

*Note:* Differences are considered statistically significant at *p* < 0.05.

Abbreviations: M, median; max, maximum; min, minimum.

**Table 3:** Comparison of clinical parameters between the navigation‐assisted surgery and conventional surgery groups.

	**Navigation-assisted surgery group (** **n** = 140**)**	**Conventional surgery group (** **n** = 48**)**	**Mann–Whitney** **U** **test result**	**Z**	**p** **value**
Hospitalization days, M (min–max)	9.00 (3.00–23.00)	11.00 (5.00–20.00)	2753.00	−1.87	0.06
Preoperative days, M (min–max)	5.00 (1.00–21.00)	6.00 (1.00–13.00)	2982.50	−1.17	0.24
Postoperative days, M (min–max)	4.00 (1.00–16.00)	4.50 (2.00–15.00)	2620.00	−2.30	0.02
Operative time, M (min–max) (minutes)	250.00 (50.00–730.00)	180.00 (90.00–570.00)	2213.00	−3.53	≤ 0.001

*Note:* Differences are considered statistically significant at *p* < 0.05.

Abbreviations: M, median; max, maximum; min, minimum.

**Figure 3:**
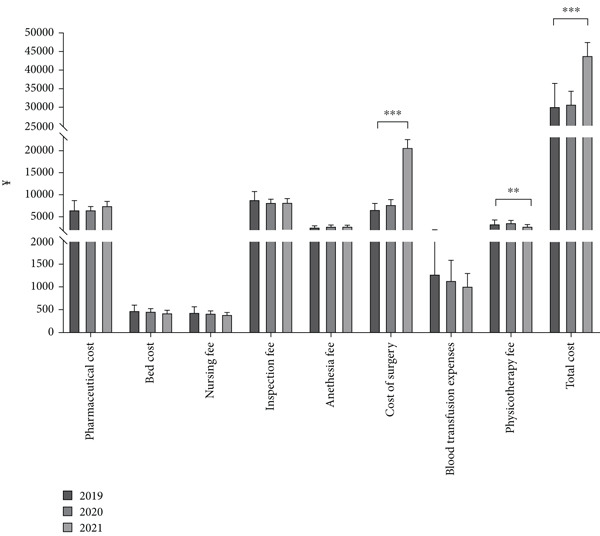
Comparison of economic parameters in the navigation‐assisted surgery group over time.  ^∗∗^ *p* < 0.01.  ^∗∗∗^ *p* < 0.001.

**Figure 4:**
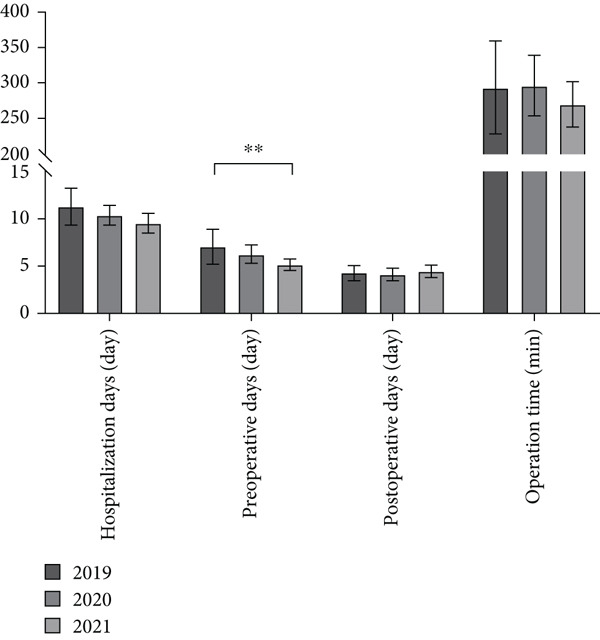
Comparison of clinical parameters in the navigation‐assisted surgery group over time.  ^∗∗^ *p* < 0.01.

In the subgroup analysis stratified by year, 21, 50, and 69 patients underwent navigation‐assisted operations in 2019, 2020, and 2021, respectively (Table 4). In the conventional surgery group, 40 and eight patients underwent surgery in 2017 and 2018, respectively. In the navigation‐assisted surgery group, more patients were treated by sacroiliac screw fixation+Stoppa approach over time (2019 vs. 2020 vs. 2021, 52.38% vs. 54.00% vs. 73.91%, *p* = 0.02), fewer patients were treated in the ICU over time (14.29% vs. 4.00% vs. 1.45%, *p* = 0.04), and more patients suffered severe injury over time (ISS ≥ 25, 4.79% vs. 4.00% vs. 14.49%, *p* = 0.02) (Table 4). In 2021, patients had a higher surgical cost (2019 vs. 2020 vs. 2021, M, ¥7374.00 vs. ¥7200.00 vs. ¥22,074.00, *p* < 0.001) and total cost (M, ¥26,469.13 vs. ¥27,216.78 vs. ¥40,970.18, *p* < 0.001), whereas in 2019, patients had a lower physiotherapy fee (M, ¥3230.00 vs. ¥3507.00 vs. ¥2371.50, *p* = 0.02) and shorter preoperative hospital stay (M, 6 vs. 5 vs. 4 days, *p* = 0.03, Figures 3 and 4).

**Table 4:** Results of subgroup analysis stratified by year for the navigation‐assisted surgery group.

	**2019 (** **n** = 21**)**	**2020 (** **n** = 50**)**	**2021 (** **n** = 69**)**	**χ** ^2^	**p** **value**
Sex, female, *n* (%)	8 (38.10)	27 (54.00)	26 (37.68)	3.44	0.18
Age (years), mean ± SD	45.90 ± 14.10	47.88 ± 14.26	48.22 ± 14.42		0.87
Surgical method				14.83	0.02
Sacroiliac screw fixation+ORIF (Stoppa approach), *n* (%)	11 (52.38%)	27 (54%)	51 (73.91%)		
Sacroiliac screw fixation+ORIF (Kocher–Langenbeck approach), *n* (%)	0 (0)	2 (4)	1 (1.45)		
Sacroiliac screw fixation+Infix, *n* (%)	9 (42.86)	13 (26)	16 (23.19)		
Sacroiliac screw fixation+ORIF (ilioinguinal approach), *n* (%)	1 (4.76)	8 (16)	1 (1.45)		
ICU treatment				6.48	0.04
Yes, *n* (%)	3 (14.29)	2 (4.00)	1 (1.45)		
No, *n* (%)	18 (85.71)	48 (96)	68 (98.55)		
Injury severity score				12.17	0.02
< 16, *n* (%)	8 (38.10)	34 (68.00)	29 (42.03)		
≥ 16, *n* (%)	12 (57.14)	14 (28.00)	30 (43.48)		
≥ 25, *n* (%)	1 (4.76)	2 (4.00)	10 (14.49)		
Pharmaceutical cost, M (min–max), ¥	5493.68 (2489.55–23,127.08)	5630.67 (3112.64–15,169.93)	6421.39 (1930.63–20,338.02)		0.13
Bed costs, M (min–max), ¥	380.00 (40.00–1180.00)	400.00 (200.00–1000.00)	400.00 (90.00–1200.00)		0.50
Nursing fees, M (min–max), ¥	355.00 (38.00–1231.00)	365.00 (162.00–1036.00)	328.00 (103.00–1030.50)		0.44
Inspection fee, M (min–max), ¥	7936.45 (1855.50–22,181.15)	7108.25 (3582.00–19,087.35)	7288.50 (3864.00–22,554.00)		0.72
Anesthesia fee, M (min–max), ¥	2320.00 (1680.00–4965.85)	2410.00 (1720.00–6330.00)	2210.00 (1480.00–6550.00)		0.45
Cost of surgery, M (min–max), ¥	7374.00 (480.00–11,756.00)	7200.00 (2530.00–22,194.00)	22,074.00 (5310.00–33,418.00)		≤ 0.001
Blood transfusion expenses, M (min–max), ¥	920.00 (0–6960.00)	420.00 (0–6540.00)	620.00 (0–4560.00)		0.94
Physiotherapy fee, M (min–max), ¥	3230.00 (186.00–8040.00)	3507.00 (688.00–8760.00)	2371.50 (783.00–6652.00)		0.02
Total cost, M (min–max), ¥	26,469.13 (8825.05–76,559.23)	27,216.78 (18,912.67–74,123.71)	40,970.18 (17,972.24–77,941.02)		≤ 0.001
Hospitalization days, M (min–max)	10.00 (7.00–23.00)	9.50 (5–19)	9.00 (3–24)		0.13
Preoperative days, M (min–max)	6 (3–21)	5 (2–15)	4 (1–14)		0.03
Postoperative days, M (min–max)	4 (2–8)	3 (1–10)	4 (1–16)		0.77
Operative time, M (min–max), minutes	240 (85–615)	277.50 (50–640)	240 (60–730)		0.59

*Note:* Differences are considered statistically significant at *p* < 0.05.

Abbreviations: M, median; max, maximum; min, minimum.

**Figure 5:**
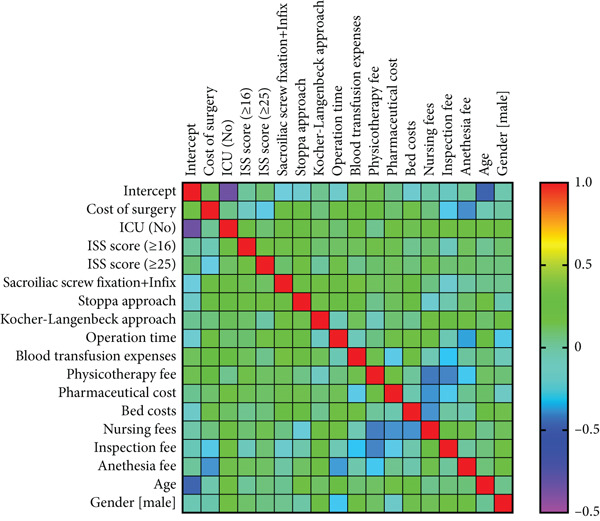
The heat map of correlations between total cost and the included parameters. The closer the color is to red, the stronger the positive correlation; the closer the color is to purple, the stronger the negative correlation.

Results of the multivariate analysis showed that total cost was positively correlated with all economic parameters, among which the positive correlations with surgical cost and ICU treatment were statistically significant (*p* < 0.001); however, total cost had no significant correlation with ISS, surgical method, operation duration, age, or gender (*p* > 0.05, Figure 5 and Table 5).

**Table 5:** Results of the correlations between total cost and the included parameters by multiple linear regression.

	**Estimate**	**Standard error**	**95% CI (asymptotic)**	**p** **value**
Intercept	309.0	88.31	134.7–483.3	≤ 0.001
Cost of surgery	0.9983	0.001536	0.9953–1.001	≤ 0.001
ICU (no)	−257.6	59.56	−375.2 to −140.0	≤ 0.001
ISS (≥ 16)	31.67	24.36	−16.42 to 79.76	0.1954
ISS (> 25)	24.18	42.70	−60.12 to 108.5	0.5719
Sacroiliac screw fixation+Infix	24.32	28.32	−31.59 to 80.23	0.3917
Sacroiliac screw fixation+ORIF (Stoppa approach)	−14.09	38.67	−90.43 to 62.25	0.7161
Sacroiliac screw fixation+ORIF (Kocher–Langenbeck approach)	−3.409	77.10	−155.6 to 148.8	0.9648
Operation time	−0.1023	0.09940	−0.2985 to 0.09390	0.3048
Blood transfusion expenses	1.012	0.01150	0.9895–1.035	≤ 0.001
Physicotherapy fee	0.9936	0.01110	0.9717–1.016	≤ 0.001
Pharmaceutical cost	0.9949	0.003701	0.9876–1.002	≤ 0.001
Bed costs	1.076	0.06257	0.9528–1.200	≤ 0.001
Nursing fees	0.9870	0.06757	0.8536–1.120	≤ 0.001
Inspection fee	0.9904	0.005383	0.9797–1.001	≤ 0.001
Anesthesia fee	1.046	0.01500	1.016–1.075	≤ 0.001
Age	−0.7568	0.7614	−2.260 to 0.7461	0.3216
Gender (male)	−27.17	22.96	−72.49 to 18.16	0.2384

*Note:* Differences are considered statistically significant at *p* < 0.05.

## 4. Discussion

In the medical field, particularly orthopedics, a sharp increase in the use of robotic navigation systems has been observed [15]. Because of their considerable enhancements in surgical safety and screw placement accuracy, robotic navigation systems have been widely used in the process of placing long sacral screws for fixation of the posterior pelvic ring [12]. Over the past few years, the high financial burden imposed by robotic navigation systems has been described in the gynecological [13, 14] and orthopedic fields [16, 17], which has raised growing concerns regarding their cost‐effectiveness. In our study, 278 patients were included to compare the cost‐effectiveness of conventional surgery and navigation‐assisted conventional surgery for the treatment of pelvic fractures.

Our research results demonstrate that although the cost of surgery, anesthesia fee, and total cost were higher in the navigation‐assisted surgery group than in the conventional surgery group, the pharmaceutical cost and nursing fee were lower in the navigation‐assisted surgery group than in the conventional surgery group (Figure 1). The navigation‐assisted surgery group had a shorter postoperative hospital stay than the conventional surgery group; however, it had a longer operative time than the conventional surgery group (Figure 2). Recently, the advantages of the navigation system have been presented, such as lower fluoroscopy frequency and time, shorter operative time, shorter incision length, less blood loss, and shorter anesthesia time [8, 18]. Yet, those operative and anesthesia times, which contradict our results, may not cover the preparation time of the equipment [8]. Studies have also indicated that, for surgeons with a lower volume of operations, robot‐assisted systems may lead to an increase in surgical time [19]. Furthermore, more time may be needed for tracer installation, preparation of special surgical instruments, device connections, and removal of the patient′s surgical gown to plan the sacroiliac screw path. A prospective randomized comparison revealed that excellent and good screw placement rates in the robot‐assisted group were superior to those in the freehand group. The number of guidewire attempts in the robot‐assisted group was less than that in the freehand group [20], which protects patients from potential harm, for example, multiple false passages, nerve root damage, and multiple drill holes. From this viewpoint, these are the reasons why patients in the navigation‐assisted surgery group had a shorter recovery time in the hospital and lower drug and nursing care costs. The cost of surgery and total cost of the navigation‐assisted surgery group were significantly higher than those of the conventional surgery group, which is consistent with the findings of other studies [13, 14, 16, 17]. The equipment usage fee was a significant factor contributing to higher costs in the navigation‐assisted surgery group. In our center, patients have to pay ¥6000.00 per time for the use of the robot that has been included in the cost of surgery and total cost because the hospital has to balance the annual maintenance cost of the Tinavi navigation‐assisted robot (¥700,000/year), fee of disposable (cost of sterilization, ¥200/time), and the financial income. The high costs of purchasing and maintaining the navigation system and the need for sterilization of special surgical instruments and equipment during the operation were reported as the main reason for the expensive fee of the navigation‐assisted procedure [21]. However, despite these higher costs, navigation‐assisted surgery may result in greater economic benefits in the long term. For instance, because navigation‐assisted surgery can provide more precise operations than conventional surgery, it can reduce high surgical efficiency and less radiation‐exposure time, thereby reducing additional costs [18, 22–24]. Moreover, because navigation‐assisted surgery can shorten recovery time after surgery, it can reduce patient hospitalization costs and nursing fees. Notably, the significant increase in surgical costs in 2021 indeed led to the rise in total costs (Figure 3), rather than differences in ISSs between 2021 and 2019/2020. As the multivariate statistical analysis showed that surgical costs and ICU treatment showed a positive correlation with total costs, the ISS and surgical methods showed no significant correlation with total costs (Table 5 and Figure 5). The reason for the increasing cost of surgery is that the surgical billing method was revised since 2021 due to the high maintenance costs of the navigation robot: The fee of ¥6000 was charged for each screw placed via the navigation system for patients with pelvic fractures, with a maximum robot usage fee of ¥18,000 per patient.

In the subgroup analysis stratified by year, we noted that, over time, patients undergoing navigation‐assisted surgery seemed to increasingly undergo sacroiliac screw fixation with the Stoppa approach. Furthermore, fewer patients required treatment in the ICU, and more patients had severe injuries over time (Table 3). This could be because of advancements in technology and the accumulation of physician experience, leading to the use of navigation‐assisted surgery to treat more complex and severe fractures. On the basis of these findings, we can infer that robotic assistance during surgery has several positive outcomes. First, it appears to minimize the impact of surgery on patients, leading to shorter waiting times before surgery [25]. Second, the reduced need for physical therapy and ICU treatment suggests that patients experience less severe surgical effects when surgery is assisted by robots. However, it is worth noting that compared to previous years, in 2021, both the overall patient and surgical costs significantly increased, possibly indicating a higher severity of ISS within the patient group (Table 3). Interestingly, despite these changes, the duration of surgery was not significantly different over time. This lack of change may be attributable to the longer learning curve associated with robotic system operation [26].

### 4.1. Limitations

First, because this study only included patients from the northwest region of China, the generalizability of the results is limited. Second, this was a retrospective study, which may have been subject to information bias and confounding variables. Additionally, we only considered economic burden indicators; comparisons of other aspects, such as surgical accuracy and postoperative complications, were not included in this study. In future research, it is necessary to incorporate studies from more regions and larger sample sizes to further evaluate the economic burden of navigation‐assisted surgery compared with conventional surgery in patients with Tile Types B and C pelvic fractures, taking into account the comprehensive effects of postoperative outcomes and complications. Furthermore, other factors related to navigation‐assisted surgery, for example, surgical accuracy, surgical time, and patient satisfaction, should be explored to assess its clinical value more comprehensively.

In conclusion, the application of orthopedic navigation systems in pelvic fractures represents a promising option for emerging orthopedic devices. Despite the higher costs associated with navigation‐assisted surgery, the proportion of pharmaceutical costs and nursing fees, as well as the shorter postoperative recovery time, may have a positive impact on the overall economic burden for patients. As technology evolves and becomes widely adopted, with increased proficiency among physicians, surgical costs related to anesthesia fees and equipment usage are expected to decrease. This may lead to a reduction in the economic burden on patients. Further research is required to confirm these findings.

## Consent

The authors have nothing to report.

## Disclosure

The authors used STROBE guidelines for this research when preparing and submitting the manuscript to maximize transparency and reproducibility.

## Conflicts of Interest

The authors declare no conflicts of interest.

## Funding

This study was funded by the Social Development Foundation of Shaanxi Province (2017SF‐011), the Natural Science Basic Research Program of Shaanxi (2024JC‐YBQN‐0967), and the General Program Foundation of Shaanxi Province (2019JM‐507).

## Supporting information


**Supporting Information** Additional supporting information can be found online in the Supporting Information section. The completed STROBE checklists were provided in the supporting information.

## Data Availability

The data that support the findings of this study are available from the corresponding authors upon reasonable request.
